# Remembering George L. Wied, M.D., February 7, 1921-July 25, 2004

**DOI:** 10.1186/1742-6413-2-2

**Published:** 2005-02-08

**Authors:** Dorothy L Rosenthal

**Affiliations:** 1 The Johns Hopkins School of Medicine, Baltimore, Maryland, USA

## Abstract

A personal tribute to George L. Wied, M.D., a founder of the medical subspecialty, cytopathology, who died July 25, 2004.

## Tribute

I arrived in Chicago last November for the ASC meeting, and reflexively reached for the phone to let Dr. Wied know that I had arrived, just as I had for each of countless trips to Chicago over the past 25 years. Then I remembered that he was no longer here, having passed away in Salzburg, Austria, on July 25th. But as the meeting unfolded, I realized that George's mark was deeply imprinted on the entire scientific program, and that his legacy was carried by most of the people in attendance.

George Papanicolaou is attributed with the discovery of the cytologic method for detecting epithelial tumors. His laboratory at Cornell Medical Center in New York was the culture medium for the discipline, instructing such notable cytologists as Leopold Koss and George Wied. The medical specialty itself, however, owes its success to George Wied, for without him, it is doubtful whether it would have survived the skepticism of most other medical specialists.

Dr. Wied had faith in the importance of cytology that was unwavering and his conviction was transmitted to all who followed him around the globe. A survivor of Nazi Germany, he was a fierce defender of individual freedoms, and he translated that zeal into inclusion of all peoples in his vision. He was an instinctual teacher and taught those around him to convey the criteria of those strange cellular samples via the Tutorials of Cytology. His attention to detail was impeccable, a trait that he insisted his faculty emulate.

The TOC faculty quickly became internationally recognized experts, and the Tutorials were a successful enterprise for over 40 years. Until cytopathology became a mandatory part of the curriculum of U.S. pathology training programs, the Tutorials were among the few opportunities for physicians to become facile with the discipline. Repeat enrollment was common, as the specialty became more complex and its importance became apparent to the medical profession. For many nations TOC remained the sole source of first-hand and first-rate cytology education. Judging by the multinational backgrounds of presenters at the ASC meeting, and the excellence of the papers, Dr. Wied's goal has most assuredly been achieved.

Setting and maintaining a level of excellence was a responsibility that Dr. George Wied eagerly assumed, and expected the rest of us to uphold. It was easy to follow his example, for his charisma infused us with commitment to those eager to learn. In order to validate the professionals involved in the practice, he established the examinations of the International Academy of Cytology. He personally traveled to every venue where the exam was held, usually following either a Tutorial or International Congress of the IAC. He savored each opportunity to bring another disciple into the fold of cytology.

But where Dr. Wied had the most fun was in the realm of cytology automation. As I listened Sunday to the presentations of experiences with the "new" imaging systems and their integration into the clinical cytology laboratory, I recalled the numerous conferences devoted to development of computerized scanners. As early as 1951, Dr. Wied recognized that computers would become an essential part of our daily lives, for data management and communication. He also realized that the task of screening slides was work intensive, subjective, and fraught with opportunities for error. If Dr. Wied didn't have the knowledge himself, he immediately reached out to others who did, and drafted them to the cause of automating his specialty. One of those recruits was Peter Bartels, a gifted optical scientist and incredibly creative problem solver. Together they built a team of researchers at the University of Chicago that attacked each obstacle to success like an army of dragon slayers.

Rather than seek the glory of discovery alone, Dr. Wied's generous spirit inspired him to organize meetings to discuss problems common to automation, bringing scientists from a variety of disciplines and numerous countries. If a potentially important contributor to the meeting did not have funding to attend, Dr. Wied would offer to support them, often personally. He was most supportive of young scientists and mentored them through their careers. These were usually pleasant meetings, as Dr. Wied's gentle and considerate nature didn't allow personal bickering. But he loved scientific controversy and he encouraged us to challenge each other to the next level of achievement. He fully believed that what was good for one research group was good for the entire profession. He was most distressed when the commercialization of automated scanners led to open fighting among the companies trusted to develop the fruits of so many years of collegial research.

He was a frequent member of NIH study sections that reviewed research proposals in automation. He was also known to drop in unexpectedly on a research group thousands of miles from his home, just to see what was happening. He was free with his advice without being condescending. He would never knowingly offend anyone. His constant encouragement and validation of the importance of scientific discovery for this young specialty was manifested through the two journals he founded and edited, *ACTA Cytologica *in 1957 and *Analytical and Quantitative Cytology and Histology *in 1979.

Recognition for his accomplishments came frequently and from various sources, including an Outstanding Investigator Award from the NIH. But if you were to reach into the heart of George Wied, I wager that he felt that his greatest accomplishment was being father to Kazutaka (George) Wied, son of his wife, Kay. An unlikely parent, Dr. Wied soon became a master at constructing dioramas out of shoe boxes, holding flash cards with German vocabulary words, and generally encouraging young George to treasure his education. Being a musician himself, Dr. Wied patiently tolerated the squawks and squeaks of his young son's efforts to be a violinist. As young George became more accomplished, after dinner performances soon became an expected treat whenever the occasion allowed.

Dr. Wied and Kay continued to travel the world even though Dr. Wied's health was becoming increasingly fragile. His final trip could have been written by a romantic novelist. He accepted a speaking engagement in Japan, but instead of a simple round trip to Tokyo, he insisted on an around-the-world ticket, "because it's cheaper!" For one of the stops, he chose to go to Vienna, a city that he loved, to once more savor the delicious schnitzel at the Intercontinental Hotel, site of the Vienna Tutorials. Kay realized that the Salzburg Music Festival was nearby and would be a good place to relax and hear the music that Dr. Wied loved so dearly. Young George, having just graduated from Stanford University, was with them, not always the case. They spent the day at numerous concerts in Salzburg, all dedicated to Czech composers, an uncanny coincidence since Dr. Wied was born in what was then Czechoslovakia. After the concerts, Kay, young George and Dr. Wied spent the evening together, remarking on the beauty of the music. Dr. Wied died in the night, with his beloved family nearby, and across the street from the home of his most admired composer, Wolfgang Amadeus Mozart.

There is an old adage, that when a loved one dies, you mourn each time for all the roles they played in your life. If it is a parent or sibling, you mourn once. However, if the person has been multiple influences in your life, you will mourn for each role. For Dr. Wied, many of us will mourn multiple times. For me, he was a teacher, mentor, advisor, friend, and role model. I will mourn many times, but will be comforted by having known him. I have already and will continue to repay his influence by transmitting his ideals to those who follow. His is truly a legacy that deserves to be perpetuated. George Wied was a man of rare breadth and depth, the kind of professional and human for which mankind will eternally thirst.

**Figure 1:**
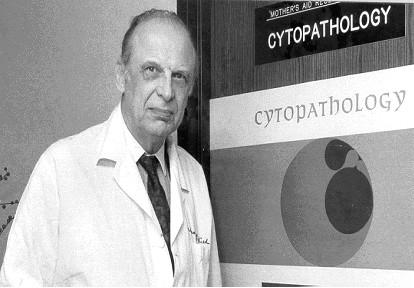
Dr. Wied at the University of Chicago Cytopathology Laboratory, circa 1990.

